# Graft immaturity and safety concerns in transplanted human kidney organoids

**DOI:** 10.1038/s12276-019-0336-x

**Published:** 2019-11-28

**Authors:** Sun Ah Nam, Eunjeong Seo, Jin Won Kim, Hyung Wook Kim, Hong Lim Kim, Kyuryung Kim, Tae-Min Kim, Ji Hyeon Ju, Ivan G. Gomez, Kohei Uchimura, Benjamin D. Humphreys, Chul Woo Yang, Jae Yeon Lee, Jin Kim, Dong Woo Cho, Benjamin S. Freedman, Yong Kyun Kim

**Affiliations:** 1 Cell Death Disease Research Center, College of Medicine, The Catholic University of Korea, Seoul, Korea; 2 Department of Internal Medicine, College of Medicine, The Catholic University of Korea, Seoul, Korea; 3 Integrative Research Support Center, College of Medicine, The Catholic University of Korea, Seoul, Korea; 4 Cancer Research Institute, College of Medicine, The Catholic University of Korea and Department of Medical Informatics, College of Medicine, The Catholic University of Korea, Seoul, Korea; 5 Department of Biomedicine & Health Sciences, College of Medicine, The Catholic University of Korea, Seoul, Korea; 6 Catholic iPSC Research Center, and Division of Rheumatology, Department of Internal Medicine, College of Medicine, The Catholic University of Korea, Seoul, Republic of Korea; 7 Division of Nephrology, Kidney Research Institute, and Institute for Stem Cell and Regenerative Medicine, Department of Medicine, University of Washington School of Medicine, Seattle, WA USA; 8 Division of Nephrology, Department of Medicine, and Department of Developmental Biology, Washington University School of Medicine, St. Louis, MO USA; 9 Department of Mechanical Engineering, Pohang University of Science and Technology (POSTECH), Pohang, Kyungbuk South Korea

**Keywords:** Induced pluripotent stem cells, Regeneration

## Abstract

For chronic kidney disease, regeneration of lost nephrons with human kidney organoids derived from induced pluripotent stem (iPS) cells is proposed to be an attractive potential therapeutic option. It remains unclear, however, whether organoids transplanted into kidneys in vivo would be safe or functional. Here, we purified kidney organoids and transplanted them beneath the kidney capsules of immunodeficient mice to test their safety and maturity. Kidney organoid grafts survived for months after transplantation and became vascularized from host mouse endothelial cells. Nephron-like structures in grafts appeared more mature than kidney organoids in vitro, but remained immature compared with the neighboring mouse kidney tissue. Ultrastructural analysis revealed filtration barrier-like structures, capillary lumens, and tubules with brush border in the transplanted kidney organoids, which were more mature than those of the kidney organoids in vitro but not as organized as adult mammalian kidneys. Immaturity was a common feature of three separate differentiation protocols by immunofluorescence analysis and single cell RNA sequencing. Stroma of transplanted kidney organoid grafts were filled with vimentin-positive mesenchymal cells, and chondrogenesis, cystogenesis, and stromal expansion were observed in the long term. Transcription profiles showed that long-term maintenance after kidney organoid transplantation induced transcriptomic reprogramming with prominent suppression of cell-cycle-related genes and upregulation of extracellular matrix organization. Our data suggest that kidney organoids derived from iPS cells may be transplantable but strategies to improve nephron differentiation and purity are required before they can be applied in humans as a therapeutic option.

## Kidney disease: “Mini-organ” transplantation needs more work

The safety and effectiveness of treating kidney disease by transplanting cultured clumps of kidney structures grown from stem cells and known as organoids need further investigation. Sun Ah Nam at The Catholic University of Korea in Seoul, with colleagues elsewhere in South Korea and the USA investigated the potential of the procedure by transplanting organoids made from human stem cells into mice. The animals were immunodeficient, thus avoiding rejection problems. The grafts generated resembled the blood-filtering structures of the kidney called nephrons, but remained insufficiently developed. Analyzing gene activity also suggested that the organoids were not adequately differentiating into the cells needed to treat disease effectively. The researchers conclude that methods to improve development and maturity of the organoids are required before organoid transplantation becomes a safe and viable treatment option in humans.

## Introduction

Chronic kidney disease (CKD) has emerged as a global healthcare crisis^1^. CKD often leads to end-stage renal disease (ESRD), for which patients require either hemodialysis or kidney transplantation in order to survive. However, both renal replacement therapies are limited—the mortality and morbidity rates in patients on dialysis remain much higher than those in the general population, while transplantation is limited by the shortage of donor organs and the need for lifelong immunosuppressive therapy. Considering the globally increasing prevalence and annual incidence of CKD, new therapeutic options are urgently needed.

CKD progression results from a loss of functional nephrons^2^. Since new nephrons cannot be generated in the adult human kidney, the functional nephrons lost during the progression of CKD cannot be recovered naturally. Therefore, regenerative medicine, in which the nephrons lost during CKD progression are replaced using stem cells, is an attractive potential therapeutic option for CKD. Recently, we and others have established several different protocols for the generation of kidney organoids from induced pluripotent stem (iPS) cells, containing segmented structures with podocytes, proximal tubules, and distal tubules in nephron-like arrangements^3–7^.

Direct comparisons with kidney tissue in vivo reveals that kidney organoids formed in vitro remain relatively immature^4^. Organoids lack bona fide vasculature, podocyte foot processes, or tubular brush borders, all of which are essential functional characteristics of the nephron^3,4^. Previous studies have shown that kidney organoids or kidney progenitor cells derived from human PS cells can be transplanted into mouse kidneys and survive in vivo, forming grafts that express markers of nephron segments and recruit vasculature from the host^3,6,8–11^.

However, it has not been clearly established whether kidney organoids transplanted into kidneys in vivo are as mature as adult kidney. As kidney organoid cultures also contain non-kidney cells (off-target cells), such as neurons, it is important to determine whether these might also pose a risk of uncontrolled proliferation or tumorigenesis^3,12,13^.

In this study, we transplant kidney organoids derived from iPS cells into mouse kidneys and focused specifically upon their maturity and safety. Although these organoids could survive in vivo, and showed some signs of maturation, the transplanted kidney organoids remained immature compared with kidney tissue in vivo. Cartilage formation reminiscent of teratoma tumors was also observed after long-term follow-up. These findings suggest caution in the application of human kidney organoids as grafts in patient populations.

## Materials and methods

### Kidney organoid differentiation

The CMC11 iPSC cell line was obtained from The Catholic University of Korea (male donor). Cells were used between passages 30 and 60. Kidney organoid differentiation was performed as described previously (Freedman et al.^3^). In brief, hPSCs were plated at a density of 5000 cells/well of a 24-well plate in mTeSR1 medium (Stem Cell Technologies)+10 µM Y27632 (LC Laboratories) on glass plates (LabTek) coated with 3% GelTrex (Thermo Fisher Scientific) (day −3). The medium was exchanged for 1.5% GelTrex in mTeSR1 (day −2), mTeSR1 (day −1), RPMI (Thermo Fisher Scientific)+12 µM CHIR99021 (Tocris) (day 0), or RPMI+B27 supplement (Thermo Fisher Scientific) (day 1.5) and cells were fed every 2–3 days to promote kidney organoid differentiation. Organoids were fixed or transplanted into NOD-SCID mice on day 18.

### Immunofluorescence and immunohistochemical analysis

For immunofluorescence, organoids were fixed on day 18, unless otherwise noted. For fixation, an equal volume of PBS (Thermo Fisher Scientific) + 8% paraformaldehyde (Electron Microscopy Sciences) was added to the medium for 15 min, after which the sample was washed three times with PBS. The fixed organoid cultures were blocked in 5% donkey serum (Millipore) + 0.3% Triton‐X‐100/PBS, incubated overnight in 3% bovine serum albumin (Sigma) + PBS with primary antibodies, washed, incubated with AlexaFluor secondary antibodies (Invitrogen), washed, and stained with DAPI or mounted in Vectashield H‐1000. For single post-embedding immunohistochemical staining (IHC), kidney and kidney organoids were embedded in wax after fixation and cut transversely at a thickness of 4 μm using a microtome. Some kidney sections and kidney organoids section were processed and stained with hematoxylin and eosin stain or Masson’s trichrome stain. Other sections were processed for post-embedding immunohistochemistry analysis. These tissue sections were hydrated with graded ethanol and rinsed in tap water. After dewaxing, the sections were incubated with retrieval solution for 10 min by microwave, washed in tap water, and incubated with methanolic H_2_O_2_ for 30 min for endogenous peroxidase blocking. Next, the sections were incubated with 0.5% Triton X-100/PBS solution for 15 min and then rinsed with PBS. Nonspecific binding sites were blocked with normal donkey serum (diluted 1:10 in PBS) for 1 h and subsequently incubated with primary antibodies overnight at 4 °C. The next day, after rinsing in PBS, the sections were incubated for 2 h in peroxidase-conjugated donkey anti-mouse or anti-rabbit immunoglobulin G (IgG; Jackson Immuno Research Lab., West Grove, PA, USA) and washed again with 0.05 M Tris buffer (pH 7.6). For detection, the sections were treated with 0.05% 3,3′-diaminobenzidine (DAB) and 0.01% H_2_O_2_. The sections were washed with distilled water, dehydrated with graded ethanol and xylene, mounted in Canada balsam, and examined by light microscopy.

For multiple post-embedding IHC, tissue and organoid sections were DAB-stained and then treated with methanolic H_2_O_2_ for 30 min to remove any peroxidase remaining from the first staining. The sections were then incubated with the other primary antibody. After washing one time with PBS, the sections were incubated for 2 h with peroxidase-conjugated donkey anti-rabbit IgG (Jackson Immuno Research Lab). For detection of peroxidase, Vector SG (Vector Laboratories, Burlingame, CA, USA) was used as a chromogen to produce a grayish blue color, which is easily distinguished from the brown staining produced by DAB. The sections were washed with distilled water, dehydrated with graded ethanol and xylene, mounted in Canada balsam, and examined by light microscopy. The following primary antibodies were used: anti-LTL (Vector Labs FL‐1321, 1:500 dilution), anti-ZO‐1 (Invitrogen 339100, 1:100), anti-NPHS1 (R&D AF4269, 1:500), anti-ECAD (Abcam ab11512, 1:100), anti-THP (MP Bio 55140, 1:200), anti-Claudin 1 (Abcam an15098, 1:100), anti-WT1 (Abcam ab89901, 1:100), anti-CD31 (R&D Systems AF3628, 1:200), anti-laminin (Sigma-Aldrich L9393, 1:200), anti-human nuclear antibody (HNA) (Merck Millipore MAB1281, 1:100), and anti-WT1 (Santa Cruz sc‐192, 1:100).

### Electron microscopy (EM) analysis

Adult mouse kidney block samples, samples from the transplanted kidney organoids and kidney organoid in vitro samples were fixed in 4% paraformaldehyde and 2.5% glutaraldehyde in 0.1 M phosphate buffer overnight at 4 °C. After washing in 0.1 M phosphate buffer, the samples were postfixed with 1% osmium tetroxide in the same buffer for 1 h at 4 °C. Next, the samples were dehydrated with a series of graded ethyl alcohol solutions, exchanged through acetone, and embedded in Epon 812.

Ultrathin sections (70–80 nm) were obtained by an ultramicrotome (Leica Ultracut UCT, Germany). The ultrathin sections were double-stained with uranyl acetate and lead citrate, after which they were examined under a transmission electron microscope (JEM 1010, Japan) at 60 kV. For quantitative analysis, 20 low-magnification (×6000) fields were randomly selected from each section of the cortex and the number of autophagosomes per 100 μm^2^ was determined.

### Analysis of kidney organoids from alternative differentiation protocols

Additional kidney organoids were differentiated using the Morizane or Takasato protocols as described in ref. ^13^. Organoids and human adult kidney were fixed in 4% paraformaldehyde (Electron Microscopy Services), cryoprotected in 30% sucrose solution overnight and embedded in optimum cutting temperature (OCT) compound (Tissue Tek). Organoids and kidneys were cryosectioned at 6 µm thickness and mounted on Superfrost slides (Thermo Fisher Scientific). Sections were washed with PBS (three times, 5 min each), then blocked with 1% bovine serum albumin (Millipore Sigma), permeabilized with 0.1% Triton X-100 in PBS and then stained with primary antibody specific for mouse anti-AQP1 (1:500, Abcam, #ab168387) and Fluorescein labeled LTL (1:500, Vector Labs, #FL-1321). Secondary antibodies included Cy3-conjugated (Jackson ImmunoResearch). Then, sections were stained with DAPI (4′,6′-diamidino-2-phenylindole) and mounted in Prolong Gold (Life Technologies). Images were obtained by confocal microscopy (Nikon C2 + Eclipse; Nikon, Melville, NY).

### Transplantation of kidney organoids derived from human iPSCs

Adherent organoids were microdissected with a 23-gauge syringe needle from 24-well plates on an inverted phase-contrast microscope, after which they were carefully transferred using a transfer pipette into an Eppendorf tube containing RB. The harvested kidney organoids in the Eppendorf tubes were transferred into PE50 tubes by slow aspiration. The organoids were isolated on day 18 of differentiation. Eight week-aged immunodeficient male NOD/SCID mice (Jackson Laboratories, West Grove, PA, USA) were used as hosts for kidney organoid transplantation. The mice were anesthetized with zoletil, after which the kidney was exposed via a dorsal flank incision. After incision of the host kidney capsule at ~2 mm with a 23-gauge syringe needle, a PE50 tube containing 10–20 kidney organoids was carefully placed under the kidney capsule. The kidney organoids were delivered by careful blowing through the other side of the PE50 tube. Mice were sacrificed at 7, 10, 14, 28, and 42 days after transplantation (*n* = 3 per each group).

To investigate the transplantation of kidney organoids into the diseased kidney, we used a UUO model. UUO was performed as previously described. Briefly, mice were anesthetized with zoletil and the left ureter was exposed via a left dorsal incision. The mid-ureter was then obstructed using a two-point ligation with silk sutures. Sham-operated mice underwent the same procedure, except that the ureter was not obstructed, and used as controls. Kidney organoids were transplanted at the time of UUO. Mice were sacrificed at 14 days after UUO. After anesthetization, the animals were perfused with phosphate buffer (PBS; pH 7.4). Next, they were fixed with 2% paraformaldehyde-lysine-periodate solution, which was administered through the heart for 10 min. After perfusion, the kidneys were removed and cut into 1–2-mm-thick slices, which were further fixed by immersion in the same fixative overnight at 4 °C. To examine the effects of kidney decellularized extracellular matrix (dECM) hydrogel kidney dECM on maldifferentiation after transplantation we transplanted kidney organoids with 1% kidney dECM under the capsule of NOD-SCID mouse kidney simultaneously. All experimental procedures were performed according to the Animal Care and Ethics Legislation and the study was approved by the Animal Care Committee of Seoul St. Mary’s Hospital.

### Decellularization of kidney

Porcine kidney tissue was sliced into 0.1−0.3-mm-thick slices and washed three times with distilled water for 30 min. Next, the slices were treated with 0.5% Triton X-100 (Sigma-Aldrich) in 1 M NaCl (Samchun Pure Chemicals) for 16 h. After then it washed three times again for 1 h. Treatment with DNase was performed to ensure removal of residual DNA from the decellularized tissue. An isotonic 1X phosphate buffered saline was prepared, and DNase was dissolved to a final concentration of 45 U/ml. The tissues were then added with DNase-containing solution at 37 °C for 6–7 h for to allow enzymatic treatment within the active period. The DNase-treated tissue slices were then washed with PBS for 12 h, followed by sterilization with 0.1% peracetic acid solution for 1 h and another washing using distilled water. Decellularized tissues were freeze-dried at −80 °C and then used for biochemical characterization and kidney dECM hydrogel preparation.

### Microarray experiment

Total RNA was prepared from the kidney organoids in vitro and the transplanted kidney organoids using TRIzol (Invitrogen). Gene expression studies were performed using the Affymetrix One Cycle labeling kit. The data were summarized and normalized with robust multi-average (RMA) method implemented in Affymetrix® Power Tools (APT). We exported the result with gene level RMA analysis and performed the differentially expressed gene (DEG) analysis. Statistical significance of the expression data was determined using LPE test and fold change in which the null hypothesis was that no difference exists among groups. False discovery rate (FDR) was controlled by adjusting *p* value using Benjamini-Hochberg algorithm. For expression profiles, the median absolute deviation was calculated, and highly variable genes were selected for hierarchical clustering with average linkage.

### Gene set enrichment analysis (GSEA)

For functional gene set-level pathway analysis, GSEA (software v3.0) was performed using the curated pathway information from MSigDB (c2cp.v6.2.symbols; 1329 gene sets; PMID: 16199517). For gene set enrichment analysis, nominal *P* values were estimated for 1000 permutations of genes for each GO category. Gene sets showing a substantial enrichment (i.e., top 10 gene sets with the highest normalized enrichment scores) either to in vitro cultured organoids compared with those transplanted or vice versa. Other parameter options for the GSEA run include type of permutation was generated by random gene sets and collapse data to gene symbols were set to FALSE.

## Results

### Nephron-like structures engraft and undergo structural changes after transplantation

**Fig. 1:**
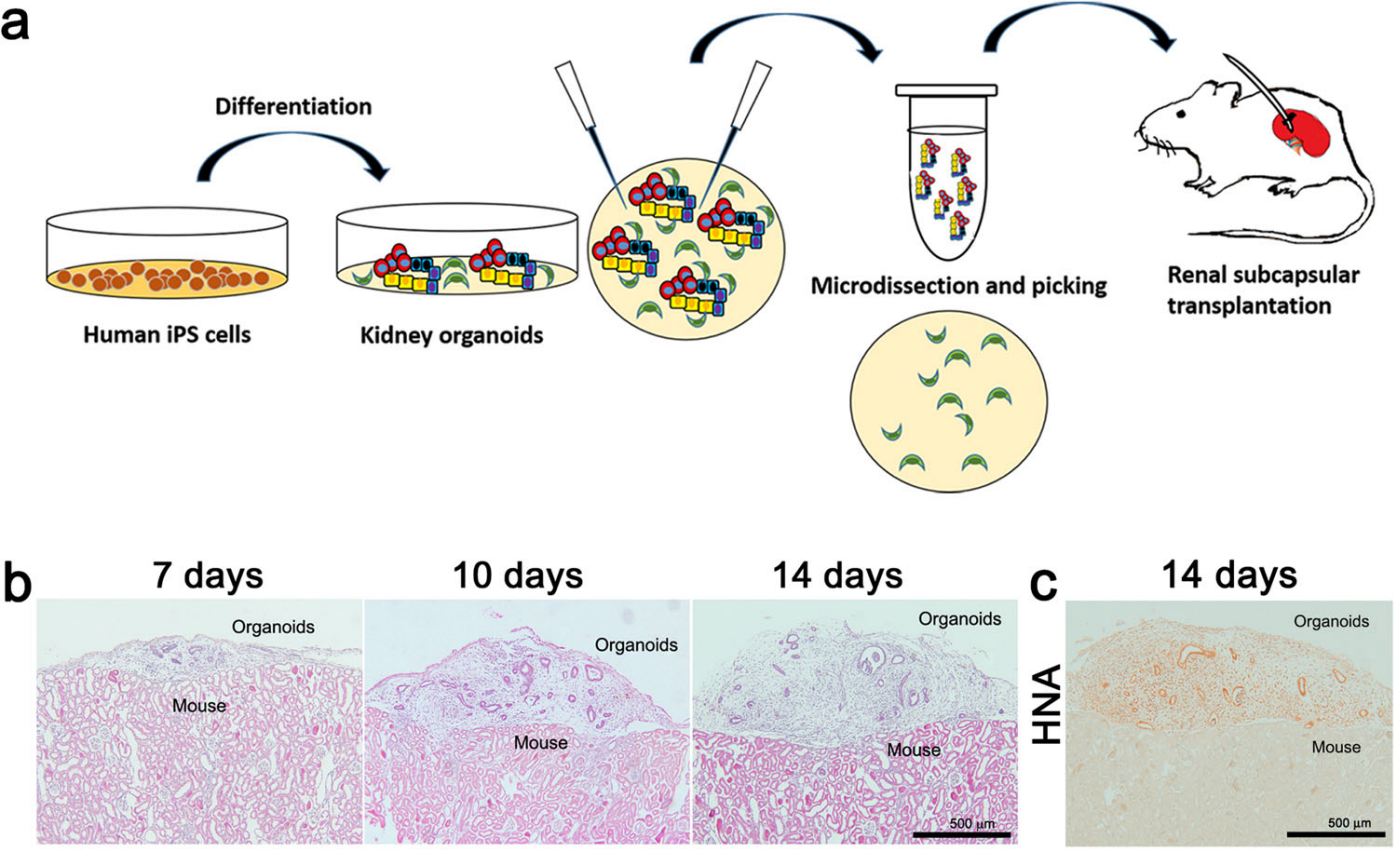
Transplanted human kidney organoids grow with nephron-like structures over time. **a** Experimental design. Kidney organoids were differentiated from human iPS cell by an adherent culture differentiation protocol and were purified by microdissection. The microdissected nephron structures were subsequently implanted beneath the kidney capsule of immunodeficient NOD-SCID mice. **b** Representative images of H&E staining after transplantation. Scale bars, 500 μm. **c** Representative images of immunohistochemical staining images with HNA after transplantation. Scale bars, 500 μm.

We began by developing a protocol to enrich grafts for nephron-specific cells away from non-kidney contaminants (e.g., neurons), which arise naturally in organoid cultures and would be undesirable for transplantation^3,12–14^. To accomplish this, we utilized an adherent culture differentiation protocol in which kidney organoids form in discrete tubular structures that can be readily purified by microdissection from the surrounding stroma^3,4,15^. We and others have shown that organoids obtained using this protocol are similar in composition to those obtained with protocols from other groups^12,14–16^. The microdissected nephron structures, which are enriched for epithelial cell types, were subsequently implanted beneath the kidney capsule of immunodeficient NOD-SCID mice for engraftment (Fig. 1a).

**Fig. 2:**
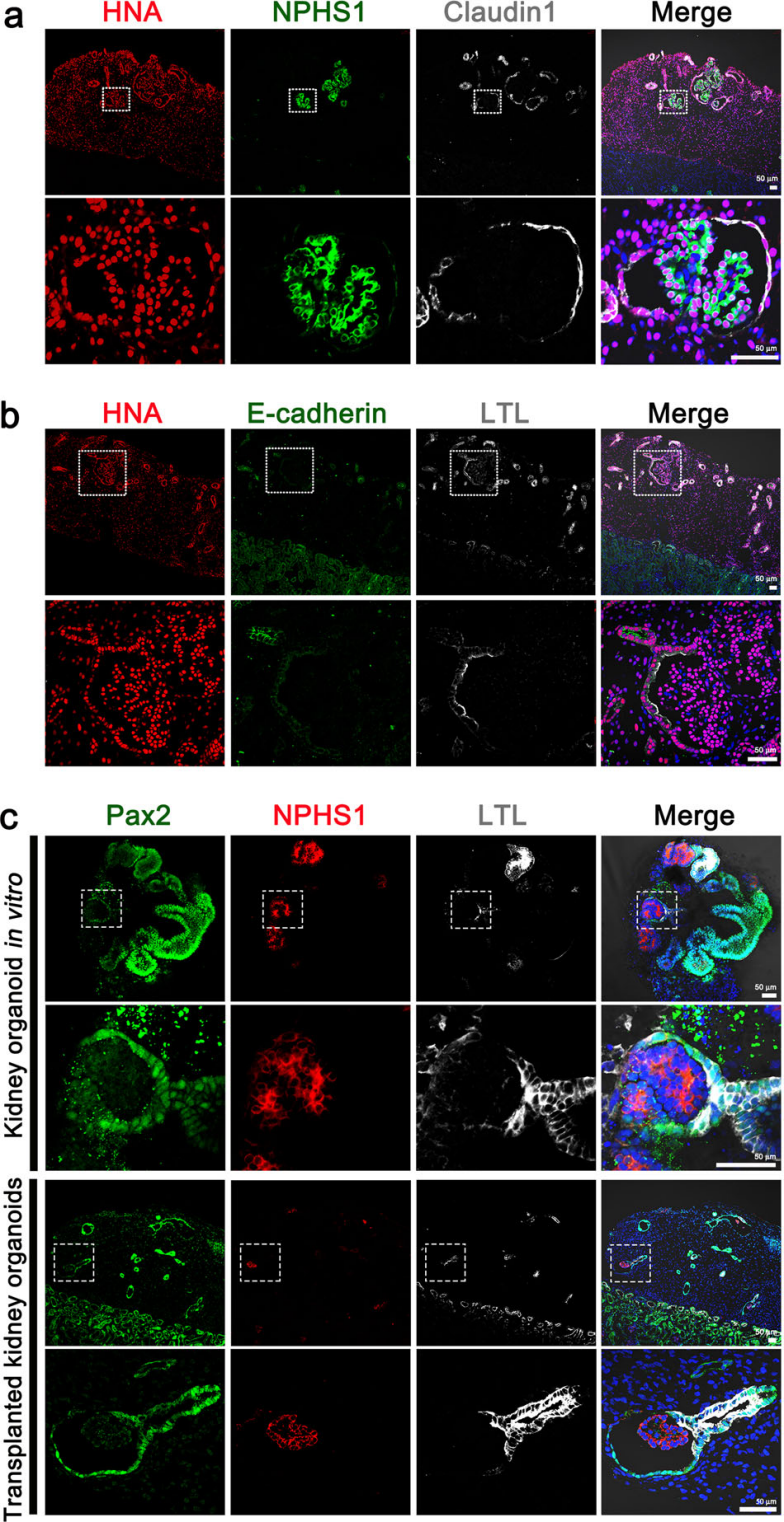
Expression of renal epithelial and progenitor cell markers in transplanted kidney organoids. **a** Representative confocal fluorescence images showing podocyte (NPHS1) and parietal epithelial cell (Claudin 1) populations in the transplanted kidney organoids. Scale bars, 50 μm. **b** Representative confocal fluorescence images showing proximal tubules (LTL) and distal tubules (E-cadherin) in the transplanted kidney organoids. **c** Representative confocal fluorescence images showing the renal progenitor cell marker, Pax2. Scale bars, 50 μm.

During the following two weeks, the tubular structures in the transplanted kidney organoids appeared to grow modestly in length and width over time, and stromal cells also expanded greatly during this time period (Fig. 1b). Human nuclear antigen (HNA) was expressed in the kidney organoid graft, but not in host mouse kidney (Fig. 1c). Immunofluorescence analysis revealed that cells expressing markers of podocytes (NPHS1), parietal epithelial cells or PECs (claudin-1), proximal tubular cells (*Lotus tetragonolobus* lectin or LTL), and distal tubules (ECAD) in the transplanted kidney organoids originated from the iPS cells, not from the host mouse kidneys (Fig. 2a, b).

We examined the structural changes of nephron-like structures after transplantation. In glomerulus-like structures, Bowman’s spaces were expanded after transplantation, compared with those of the kidney organoids in vitro (Supplementary Fig. S1A, B). In tubule-like structures, luminal spaces were greater in diameter than those of the kidney organoids in vitro (Supplementary Fig. S1C, D).

During kidney development, paired box 2 (Pax2) is expressed in the nephric duct, the mesonephric tubules, and in the metanephric mesenchyme, and is an important regulator of nephron differentiation^17,18^. As the nephron mature, Pax2 is downregulated first in the podocyte progenitor cells and subsequently in all proximal and distal tubules of the nephron^18,19^. In adulthood, very few renal differentiated cells maintain Pax2 expression^19^. We found that PAX2 was expressed in tubule-like structures and parietal epithelial cell-like structures in kidney organoid grafts, similar to kidney organoids in vitro (Fig. 2c), indicating that the nephron-like tubules in the transplanted kidney organoids graft remained immature. As an internal control, we confirmed that PAX2 was downregulated in adjacent NPHS1-positive podocytes within the graft (Fig. 2c), just as we have previously shown it to be downregulated in organoid podocytes in vitro^4^. Taken together, these findings indicate that transplantation into the host kidneys produces changes in the structure of nephron compartments in kidney organoids derived from iPS cells, but did not induce a complete maturation of the cells.

### Transplanted kidney organoids are infiltrated by endothelial cells

**Fig. 3:**
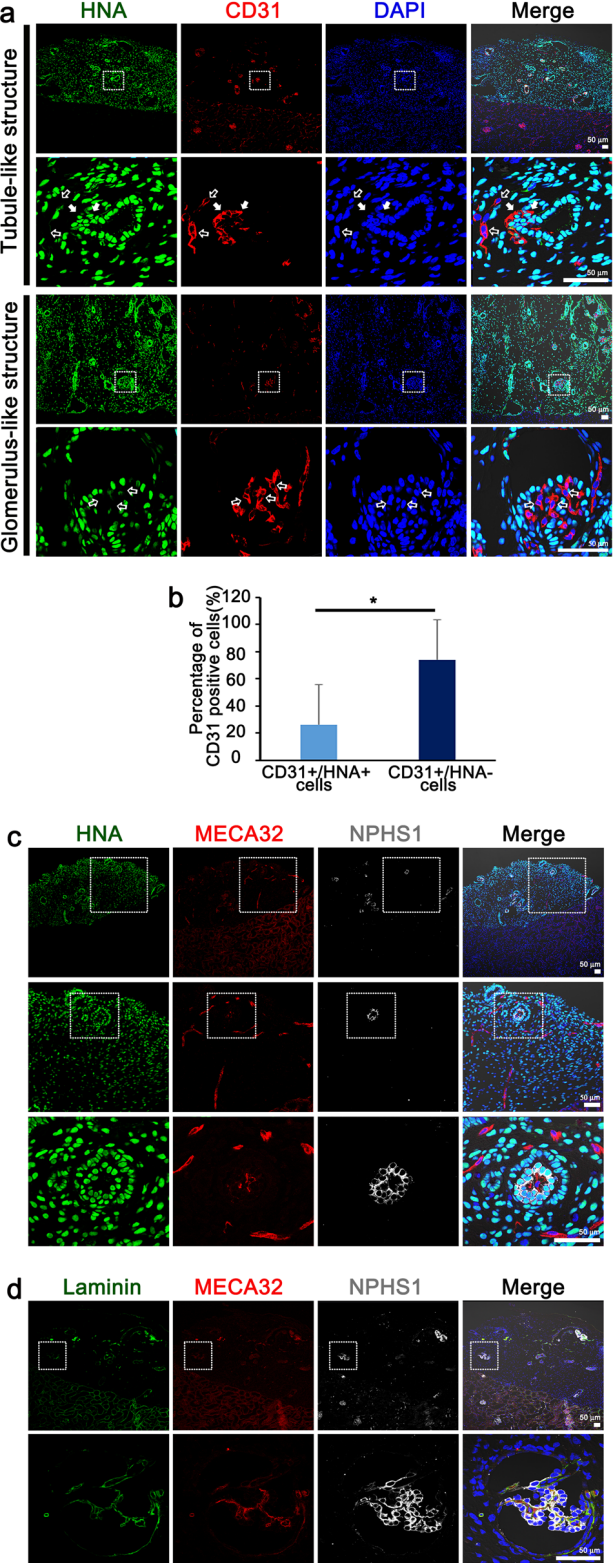
Transplanted kidney organoids are vascularized with infiltrating endothelial cells from the host kidney and form GBM-like structures. **a** Representative confocal images of transplanted kidney organoids at 14 days after transplantation. White arrow in the enlarged images indicate CD-31-positive, HNA-positive cells and open arrow indicate CD-31-positive, HNA-negative cells. Scale bars, 50 μm. **b** Quantification of CD31+/HNA+ cells and CD31+/HNA− cells of the CD31+ cells. **c** Representative confocal images of podocytes (NPHS1) and mouse endothelial cells (MECA32) in the transplanted kidney organoids at 14 days after transplantation. Scale bars, 50 μm. **d** Representative confocal images of glomerular-like structures in the transplanted kidney organoids. Cells were stained for NPHS1, MECA32 and laminin. Scale bars, 50 μm.

Vascularization is crucial for nephron maturation in kidney development. Furthermore, the vascularization of kidney metanephric grafts after transplantation is important for survival and successful engraftment^20^. Therefore, we investigated the extent of vascularization of the transplanted kidney organoids and the origin of the endothelial cells in the transplanted kidney organoids. We could observe human endothelial cells derived from the transplanted kidney organoids (CD31+/HNA+), but their proportion of the endothelial cells in the graft was small (Fig. 3a, b). Mouse endothelial cells (MECA32+) were abundantly observed in the transplanted kidney organoid graft as well as within the glomerulus-like structures (Fig. 3c), which indicates that endothelial cells from the host mouse kidney had extensively infiltrated into the transplanted kidney organoids and formed a vascular network.

The glomerular basement membrane (GBM) is essential for filtering the blood during urine production in the mammalian kidney^21,22^. Therefore we examined whether the infiltrated mouse endothelial cells express markers of GBM in the transplanted kidney organoids. The mouse endothelial cells in glomerulus-like structures co-localized with laminin, an extracellular matrix component of GBM (Fig. 3d). This suggested that a GBM-like structure may be forming in the grafts at the interface between endothelial cells and podocytes.

### Ultrastructure of podocytes and tubules reveals partial graft maturation

**Fig. 4:**
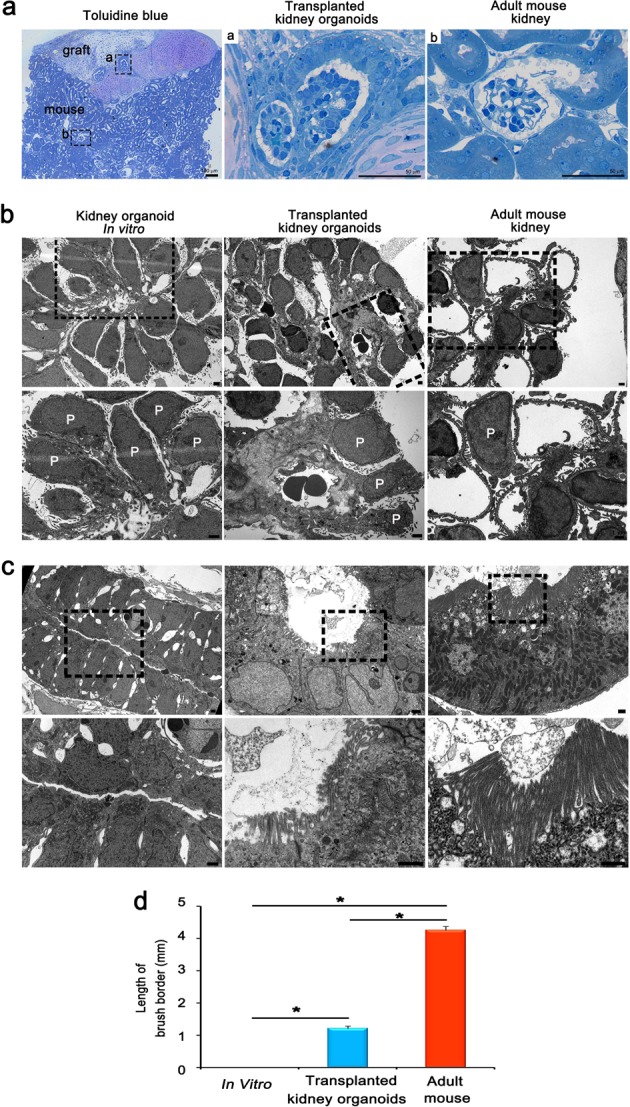
The ultrastructure of glomerulus-like structures and tubules in transplanted kidney organoids remains immature compared with adult mouse kidneys. **a** Representative images of toluidine blue staining. **b**, **c** Representative TEM images of kidney organoids in vitro, the transplanted kidney organoids and adult mouse kidney. Magnified portions of the images are shown below the images. P; podocytes. Scale bars, 1 μm. **d** Quantification of the length of brush borders (*n* = 3, kidney organoids in vitro vs. transplanted kidney organoids vs. adult mouse kidney. *p < 0.05).

We further analyzed these structures by transmission electron microscopy (TEM) to determine the cellular ultrastructure of the podocytes and tubules, and compared kidney organoids in vitro, transplanted kidney organoids, and adult mouse kidney. First, to identify the sections containing the glomerular and tubular-like structures (Fig. 4a) from the transplanted kidney organoids, toluidine blue staining was performed and sections were cut under light microscopy.

In the kidney organoids in vitro, the podocytes had apical microvilli and were arranged intermittently along poorly organized GBM‐like tracks (Fig. 4b). In contrast to these structures, red blood cell fragments were observed in the transplanted kidney organoids, suggestive of possible capillary formation (Fig. 4b). The podocytes in the transplanted kidney organoids looked similar to the organoids in vitro and direct comparison to adult kidney demonstrated that organoids lacked bona fide foot processes with well-organized tertiary interdigitations along the GBM (Fig. 4b). Glomerulus-like structures were surrounded by a tubular epithelium with intervening space, similar in architecture to a Bowman’s capsule but with a substantially thicker capsular layer (Fig. 4a, b).

Interestingly, the tubules in the transplanted kidney organoids exhibited brush borders similar to the proximal tubules of the adult mouse kidney; these borders were rarely observed in the kidney organoids in vitro (Fig. 4c). The length of brush borders, however, was shorter than that of adult mouse kidney (Fig. 4c, d). Taken together, these findings suggest that the maturation of nephron-like structures in the transplanted kidney organoids was incomplete.

### Aquaporin-1 expression in organoids is low

**Fig. 5:**
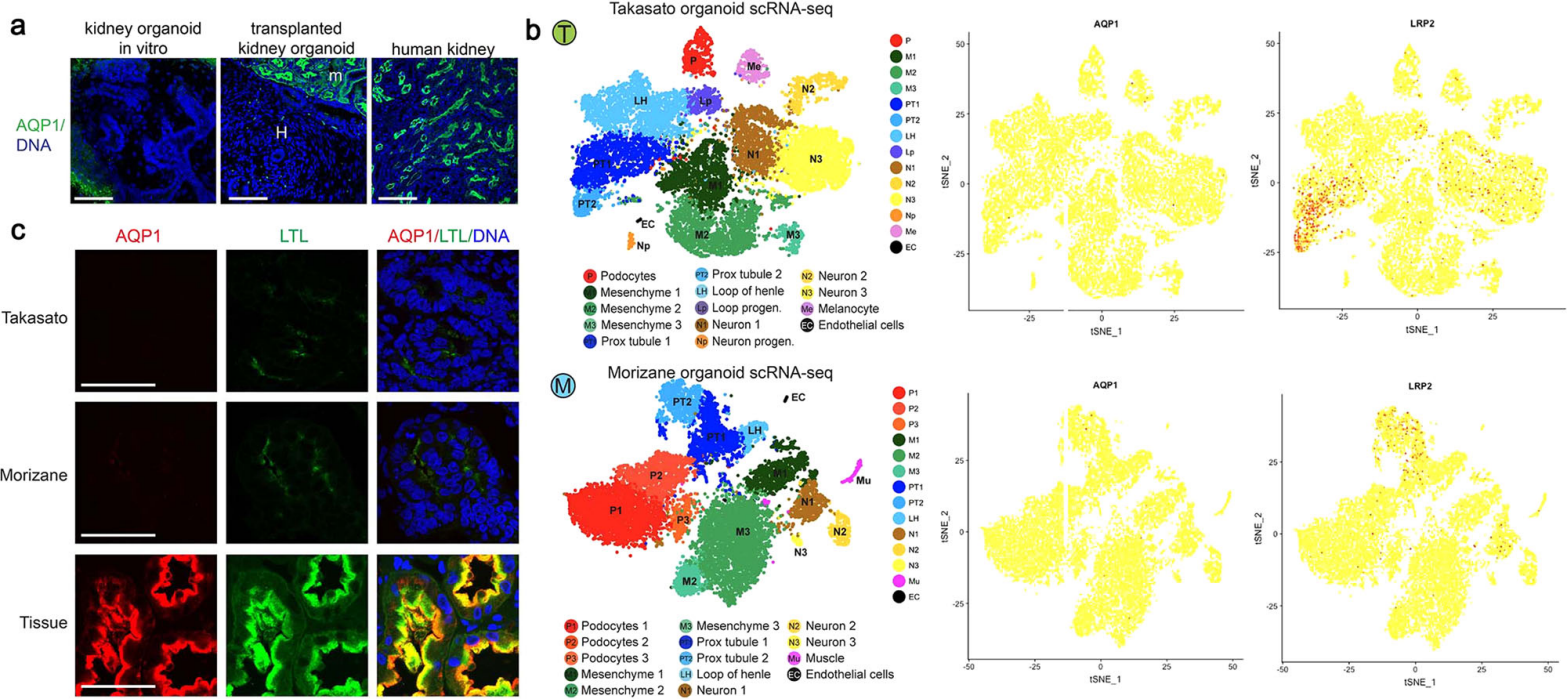
Aquaporin-1 expression is negligible in kidney organoids from different protocols. **a** Representative confocal fluorescence images of AQP1; M, mouse kidney; H, human graft. Scale bars, 100 μm. **b** t-SNE dot plots showing clusters of kidney organoid cells generated using either the Takasato (T, top) or Morizane (M, bottom) method, analyzed by single cell RNA-seq. **c** Confocal optical sections of organoids derived using these protocols or human kidney tissue, using identical laser power and gain settings for AQP1 and LTL channels. Images are representative of three different batches of organoids for each protocol. Scale bars, 50 µm.

The water channel aquaporin-1 (AQP1) is strongly expressed in kidney proximal tubules. However, we observed AQP1 to be largely absent in kidney organoid tubules in vitro, indicating these structures were not fully differentiated (Fig. 5a). In grafts, we found that AQP1 expression was likewise negligible in the human tubules (Fig. 5a). In contrast, AQP1 was robustly detected in the neighboring mouse kidney tubules, and in human kidney tissue, with same antibody (Fig. 5a).

To test whether this was a deficiency in our particular differentiation protocol, we further examined the expression of AQP1 in kidney organoids differentiated by two different (alternative) protocols^5,7^. Single cell transcriptomic analyses data revealed negligible expression of AQP1 in kidney organoid tubules differentiated by both of the alternative protocols (Fig. 5b)^13^. In contrast, LRP2 (megalin) was readily detected in tubular cells in these same datasets (Fig. 5b). Immunofluorescence analysis of proximal tubules from both of the other protocols also demonstrated negligible signal for AQP1, compared with sections of kidney tissue in vivo (Fig. 5c). Thus these organoids resembled our own. Binding of LTL was also reduced compared with tissue sections, but was nevertheless readily detected (Fig. 5c). Similar results were obtained with two additional batches of these organoids derived using the two alternative protocols (Supplementary Fig. S2). Artificially brightening the organoid images strengthened the LTL signal and suggested a possible faint signal for AQP1, but this remained substantially lower than in the images of kidney tissue, which when similarly brightened appeared severely overexposed. These findings demonstrate that lack of AQP1 is a general property of organoids differentiated using three different protocols currently in common use, suggests that the proximal tubules derived using these protocols are similar in composition, and indicates that all of these protocols generate organoids that are immature compared with those in vivo.

### Organoid grafts are progressively overgrown by stromal cells

**Fig. 6:**
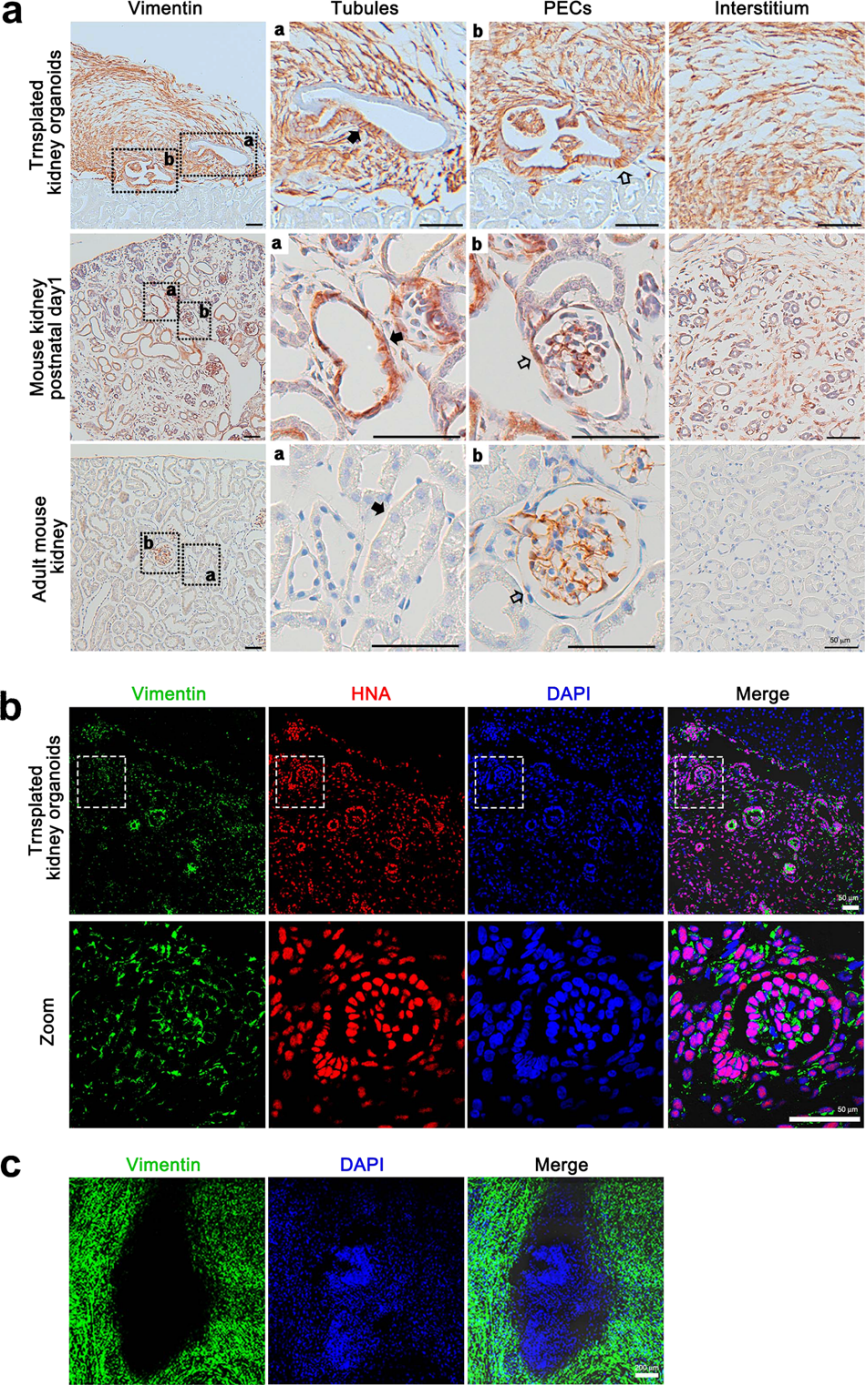
Nephron-like structures in kidney organoid grafts are not fully mature. **a** Representative immunohistochemical staining images showing vimentin staining in transplanted kidney organoids harvested at 14 days after transplantation, mouse kidneys with postnatal day 1 and adult mouse kidneys. Magnified portions of the images are shown to the right of the images. White arrows indicate parietal epithelial cells and black arrows indicate tubules. Scale bars, 50 μm. **b** Representative confocal images of HNA and vimentin in the transplanted kidney organoids at 14 days after transplantation. Scale bars, 50 μm. **c** Representative confocal images of vimentin in kidney organoid outgrowth cells 7 days after transfer onto glass plates coated with 3% GelTrex. Scale bars, 200 μm.

The transplanted kidney organoids exhibited excessive growth of stromal cells, which expressed vimentin, a mesenchymal cell marker, similar to postnatal mouse kidney, whereas interstitial cells of adult mouse kidney were negative for vimentin (Fig. 6a). The tubules and PECs-like structures in the kidney organoids also showed partial staining for vimentin (Fig. 6a), which resembled the pattern of vimentin expression in mouse kidney with postnatal day 1, whereas the tubules and PECs in adult mouse kidney were negative for vimentin staining (Fig. 6a). The persistence of vimentin in these structures suggested that maturation of nephron-like structures is not complete in the transplanted kidney organoids^23–25^.

To examine whether the vimentin-positive cells in the stroma and nephron-like structures originate from human kidney organoids or host mouse kidney, we stained vimentin and HNA simultaneously. The vast majority of vimentin-positive cells expressed HNA (Fig. 6b). We also replated purified kidney organoids onto glass plates coated with 3% GelTrex and stained vimentin on organoid outgrowth cells in vitro 7 days after plating (Supplementary Fig. S3)^15^. Abundant organoid outgrowth cells expressed vimentin (Fig. 6c, Supplementary Fig. S3). These findings suggest that the vimentin-positive cells in the stroma in these grafts derive from outgrowth cells of the transplanted kidney organoids.

### Kidney organoids are transplantable into mouse kidneys with unilateral ureteral obstruction

**Fig. 7:**
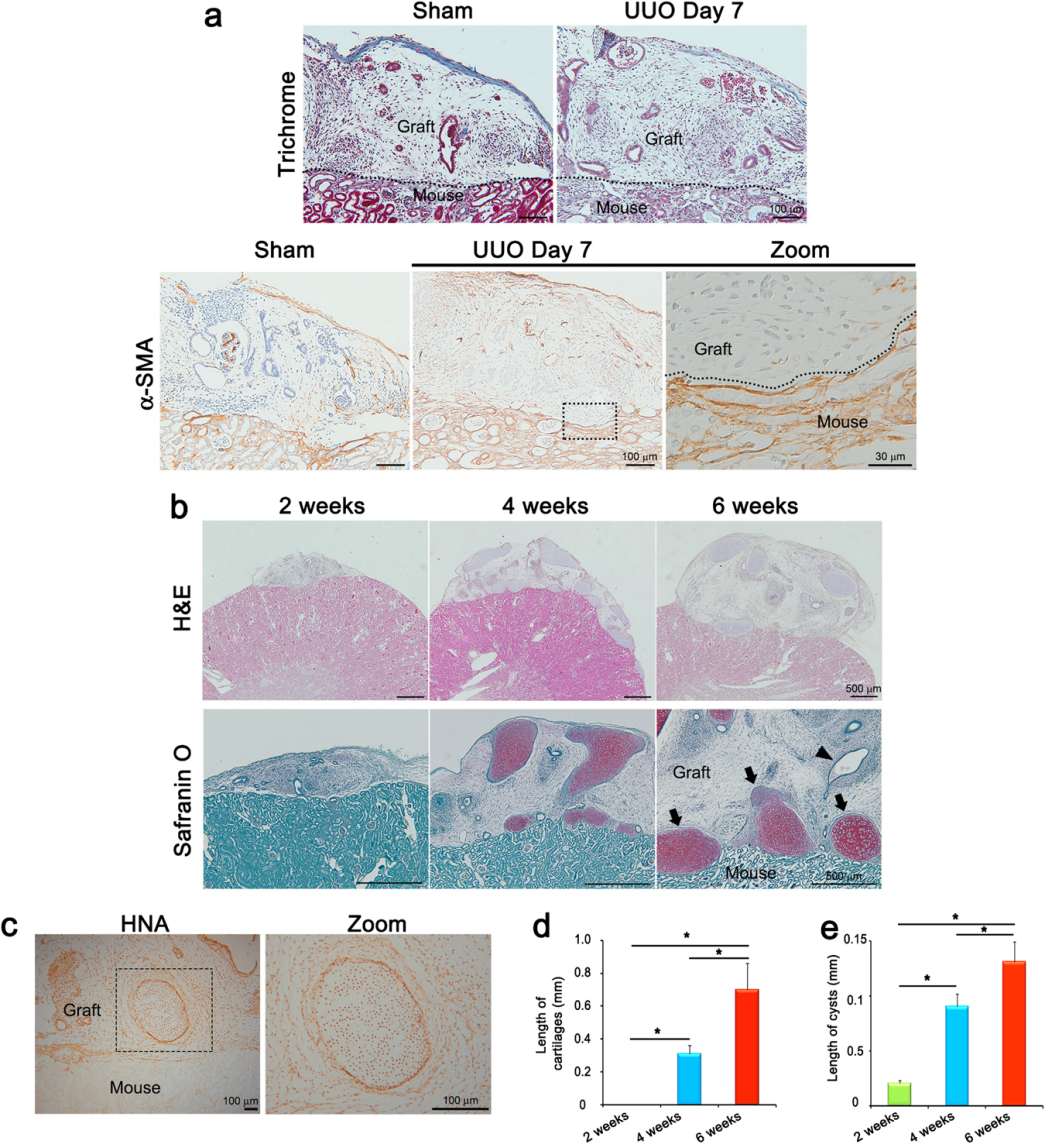
Kidney organoids are transplantable into mouse kidneys with unilateral ureteral obstruction. **a** Representative images of Trichrome staining, and immunohistochemical staining for α-SMA are shown. Cells were stained at 7 days after UUO. Scale bars, 100 μm. A zoom image shows few α-SMA positive cells in the grafts, whereas they were abundantly expressed in the neighboring mouse kidney tissue with UUO. Scale bar, 30 μm. **b** Representative images of H&E staining and immunohistochemical staining for safranin O. Arrows indicate cartilages and an arrow head indicate a cyst. Scale bars, 500 μm. **c** Representative images of immunohistochemical staining for HNA. Scale bars, 100 μm. Quantification of the length of cartilages (**d**) and cysts (**e**) (*n* = 3, 2 weeks vs. 4 weeks vs. 6 weeks after kidney organoids transplantation. *p < 0.05).

Adding nephrons to diseased kidneys with nephron loss may in the future be an effective therapeutic option. Thus, to investigate whether the transplanted kidney organoids survive and vascularize in the setting of kidney disease, we performed kidney organoid transplantation using a mouse model of unilateral ureteral obstruction (UUO), which exhibits progressive tubulointerstitial fibrosis^26^. The transplanted kidney organoids were vascularized and survived in the transplanted graft at 2 weeks after UUO and transplantation (Fig. 7a).

Interestingly, few fibrotic cells expressing smooth muscle alpha actin (SMA) were observed in the transplanted kidney organoid grafts, whereas the neighboring mouse kidney tissue with UUO had widespread tubulointerstitial fibrotic lesions (Fig. 7a). Thus, the grafts did not manifest phenotypic hallmarks of kidney disease observed in the neighboring kidney. Collectively, these studies suggest that engraftment of organoids is possible in settings of renal disease, and that grafts placed beneath the kidney capsule may be partially protected from damage to the adjacent kidney.

### Chondrogenesis and cyst formation with degenerated nephron-like structures in long-term follow-up

The transplanted kidney organoids survived until at least 6 weeks after transplantation. In the absence of UUO, cartilage formation was observed in the transplanted kidney organoid grafts at 4 weeks after transplantation (Fig. 7b). HNA was expressed in the cartilaginous masses in the transplanted kidney organoids (Fig. 7c). The cartilage became enlarged at 6 weeks after transplantation (Fig. 7b, d, Supplementary Fig. S4). Fewer nephron-like structures were observed, and cysts were formed at 4 weeks and gradually increased in size at 6 weeks after transplantation (Fig. 7b, e). The nephron-like structures appeared to be degenerated 4 weeks after transplantation and well-organized nephron-like structures were rarely observed at 6 weeks after transplantation (Fig. 7b). The presence of human cartilage inside these grafts, interspersed with the human organoid-derived mesenchyme, suggests that the cells initially transplanted included non-kidney or partially differentiated cells, even though the organoids were purified prior to transplantation. Such contaminants could pose a safety concern in humans receiving kidney organoid grafts.

Kidney decellularized extracellular matrix (dECM) hydrogel contains ECM protein such as collagen-IV, laminin, and heparan sulfate proteoglycan, and provides a microenvironment similar to normal kidney^27^. To determine the role of microenvironment provided from kidney dECM, we transplanted kidney organoids together with kidney dECM. We found that cartilage formation in the group transplanted with kidney dECM was decreased compared with the group transplanted with kidney organoids only (Supplementary Fig. S5), which suggest that maldifferentiation after kidney organoids transplantation may be corrected by adjustment of microenvironment components provided by kidney dECM.

### Altered gene expression after transplantation of kidney organoids with long-term follow-up

**Fig. 8:**
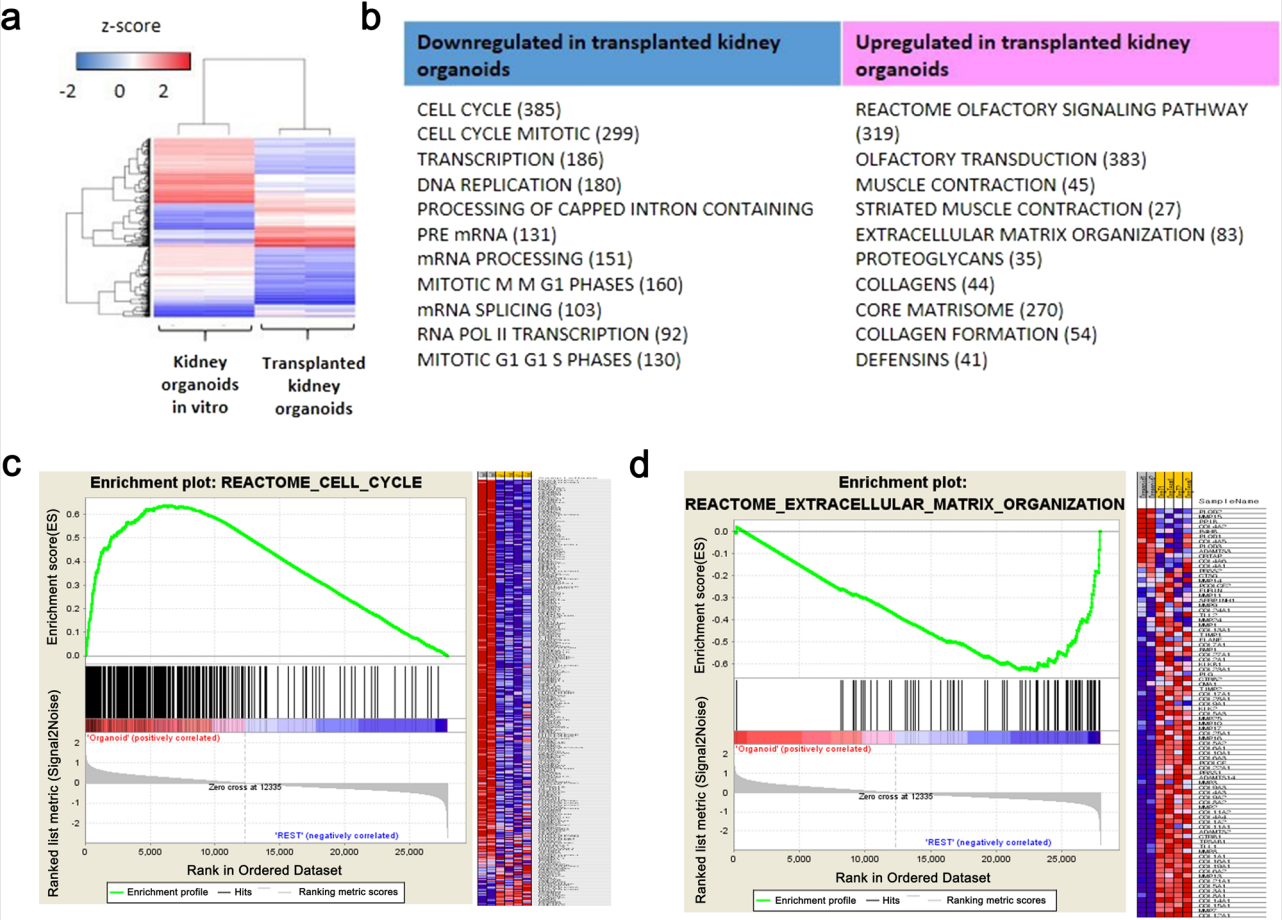
Altered gene expression after transplantation of kidney organoids with long-term follow-up. **a** Hierarchical clustering of 1000 variable probes (median absolute deviation >0.04). **b** Top 10 gene curated canonical pathway (MSigDB c2cp) terms enriched. Gene set enrichment plot of GSEA with significant downregulation (**c**) and upregulation (**d**) in the transplanted kidney organoids.

To determine the alteration of gene expression in the transplanted kidney organoids after long-term follow-up, we examined the transcriptional profiles of the transplanted kidney organoids (6 weeks after transplantation), compared with those of kidney organoids in vitro (day 18). Hierarchical clustering of highly variable genes clearly segregated the transplanted kidney organoids and kidney organoids in vitro (Fig. 8a), indicating a substantial difference in transcriptomes.

Gene set enrichment analysis was performed to identify the candidate molecular functions associated with these transcriptomic changes. Overall, 29 and 353 GO categories exhibited significant enrichment with upregulated and downregulated genes, respectively, in the transplanted kidney organoids compared with kidney organoids in vitro. Top 10 gene curated canonical pathway (MSigDB c2cp) terms enriched were shown in Fig. 8b. Of note, among GO categories downregulated in the transplanted kidney organoids, genes for “cell-cycle” and related functions (e.g., “cell cycle mitotic” and “DNA replication”) were significantly enriched. The enrichment plot of 253 genes from “cell-cycle”-related gene sets is shown with the top 20 leading-edge genes (Fig. 8c, Supplementary Table S1). In addition, among the GO categories upregulated in the transplanted kidney organoids, extracellular matrix organization-related GO function was observed (Fig. 8b, d), which is consistent with the expansion of stromal cells and cartilage in the transplanted kidney organoids. The enrichment plot of 47 genes from “extracellular matrix organization”-related gene sets is shown with the top 20 leading-edge genes (Fig. 8d, Supplementary Table S2). Taken together, these results from the transcription profiles indicate that long-term maintenance after kidney organoids transplantation may induce transcriptomic reprogramming with prominent suppression of cell-cycle-related genes and upregulation of extracellular matrix organization.

## Discussion

Adding functional nephrons derived from hPSCs is a potentially attractive therapeutic option for kidney disease with nephron loss, but is also fraught with concerns over safety and efficacy. Thus, it is essential to determine the maturity of the transplanted organoids and their capacity for maldifferentiation.

Some recent reports describe in detail vascularization of human kidney organoid grafts beneath the kidney capsule and suggest increased maturity of these structures^8–12^. Sharmin et al. first reported that the glomeruli in the transplanted spheres with human iPSC-derived nephron progenitors were vascularized with host mouse endothelial cells with further maturation although the capillary loops were rarely formed^8^. van den Berg et al. also showed that transplanted kidney organoids derived from hPSCs have the matured glomerular filtration barrier and tubular epithelium with host mouse-derived vascularization^9^. However, it is still needed to define the maturation stage in accordance with mammalian kidney development and find evidence for safety after long-term follow-up.

We also observe some vascularization of podocytes and maturation in tubules in our grafts similar with previous studies^8–12^. However, podocytes do not appear to form fully mature foot processes, and instead are decorated by apical microvilli, which are distinct structures^4^. We also present the evidence that these still lack fully mature features such as aquaporin-1 expression, compared with neighboring mouse kidney tissue. In addition, we report maldifferentiation in the transplanted graft with the formation of cartilage and cysts of increased-size after long-term follow-up.

Many of the cells of the transplanted kidney organoid graft were vimentin-positive mesenchymal cells, which may differentiate into non-renal cells such as chondrocyte, adipocytes or osteocyte^28^. We have shown previously that kidney organoids are contaminated by non-kidney cell types, including neuronal, endothelial, and muscle cells, as well as rare undifferentiated pluripotent stem cells^3,14^. We have recently confirmed the presence of non-kidney contaminants in two other protocols for kidney organoid differentiation (Morizane’s protocol and Takasato’s protocol)^13^. Our own side-by-side comparisons of our differentiation protocol and those of another lab also suggest that these produce very similar types of structures^14,15^.

In this study, we attempted to avoid non-kidney contaminants by purifying epithelial organoid structures away from surrounding stroma by microdissection. Nevertheless, cartilage formation and stromal expansion were commonplace in these organoid cultures, suggesting either the persistence of these contaminants or de-differentiation of epithelia into non-kidney lineages. This highlights the importance of improving organoid differentiation protocols to promote definitive differentiation and remove all contaminants, which could potentially form tumors at the graft site. Our experiments were performed in NOD-SCID animals which have minimal immune function and cannot reject xenogeneic implants. The stromal cells as well as cartilage appeared to be human based on antibody immunofluorescence and immunohistochemistry. Importantly, to reduce maldifferentiation, we transplanted kidney organoids with kidney decellularized extracellular matrix (dECM) hydrogel and observed a decrease in cartilage formation. These data suggest an approach for improvement of maldifferentiation after kidney organoids transplantation by adjustment of the graft microenvironment.

Maturational stage of kidney organoids may differ somewhat among the research groups because of different cell composition in kidney organoids according to protocols and inter-experimental and inter-clonal transcriptional variation in kidney organoid differentiation even with the same protocol^13,29^. This variability between kidney organoids may produce slightly different maturational stages after transplantation among the research groups. For this concern, we demonstrate that immaturity of organoids in vitro to be a common feature of three distinct differentiation protocols, as illustrated by the lack of substantial AQP1 expression in proximal tubules. This argues against any particular protocol being substantially more advanced than any other. Furthermore, we have validated the maturational state of podocytes in kidney organoids differentiated by our protocol^4^. Comparative analysis of hPSC-podocytes in kidney organoids in vitro and developing mammalian kidney revealed that hPSC-podocytes phenocopied mammalian podocytes at the capillary loop stage^4^, which is similar to the developmental stage of kidney organoids from other research groups. Our detailed analysis of AQP1 expression in organoids derived using three distinct protocols (Freedman, Morizane, and Takasato) reveals that none of these methodologies yields robust expression of this classic proximal tubular marker, and all are therefore immature compared with kidney tissue in vivo. An advanced protocol to improve the maturation of kidney organoids in vitro may contribute to enhance the maturation of kidney organoids after transplantation and utilize kidney organoids as regenerative medicine for kidney disease. In conclusion, the transplanted kidney organoids derived from human iPSCs were partially vascularized from host mouse endothelial cells and exhibited signs of increased maturity, compared with kidney organoids in vitro. However, the maturational stage of both tubules and podocytes in the transplanted kidney organoids remained incomplete, and a significant population of the cells in the grafts formed stromal growths containing non-kidney cells. Strategies for overcoming the immaturity and maldifferentiation of the kidney organoids derived from iPSCs are needed before attempting transplantation of the kidney organoids into humans as a therapeutic option for nephron loss^30^.

## Supplementary information

Supplemental material
