# Correction to: Graft immaturity and safety concerns in transplanted human kidney organoids

**DOI:** 10.1038/s12276-019-0366-4

**Published:** 2020-01-20

**Authors:** Sun Ah Nam, Eunjeong Seo, Jin Won Kim, Hyung Wook Kim, Hong Lim Kim, Kyuryung Kim, Tae-Min Kim, Ji Hyeon Ju, Ivan G. Gomez, Kohei Uchimura, Benjamin D. Humphreys, Chul Woo Yang, Jae Yeon Lee, Jin Kim, Dong Woo Cho, Benjamin S. Freedman, Yong Kyun Kim

**Affiliations:** 1 Cell Death Disease Research Center, College of Medicine, The Catholic University of Korea, Seoul, Korea; 2 Department of Internal Medicine, College of Medicine, The Catholic University of Korea, Seoul, Korea; 3 Department of Internal Medicine, College of Medicine, The Catholic University of Korea, St. Vincent’s Hospital, Suwon, Korea; 4 Integrative Research Support Center, College of Medicine, The Catholic University of Korea, Seoul, Korea; 5 Cancer Research Institute, College of Medicine, The Catholic University of Korea and Department of Medical Informatics, College of Medicine, The Catholic University of Korea, Seoul, Korea; 6 Department of Biomedicine & Health Sciences, College of Medicine, The Catholic University of Korea, Seoul, Korea; 7 Catholic iPSC Research Center, and Division of Rheumatology, Department of Internal Medicine, College of Medicine, The Catholic University of Korea, Seoul, Republic of Korea; 8 Division of Nephrology, Kidney Research Institute, and Institute for Stem Cell and Regenerative Medicine, Department of Medicine, University of Washington School of Medicine, Seattle, WA USA; 9 Division of Nephrology, Department of Medicine, and Department of Developmental Biology, Washington University School of Medicine, St. Louis, MO USA; 10 Department of Mechanical Engineering, Pohang University of Science and Technology (POSTECH), Pohang, Kyungbuk South Korea

**Keywords:** Induced pluripotent stem cells, Regeneration

**Correction to:Experimental and Molecular Medicine**

10.1038/s12276-019-0336-x published online 28 November 2019

After online publication of this article, the authors noticed an error in the Affiliation section.

The correct statement of this article should have read as below.

“Two of authors’ affiliations was misprinted. Authors' affiliations list should be corrected as follows:

Sun Ah Nam^1,2^, Eunjeong Seo^1^, Jin Won Kim^1^, Hyung Wook Kim^2,10^, Hong Lim Kim^3^, Kyuryung Kim^4,5^, Tae-Min Kim^4,5^, Ji Hyeon Ju^6^, Ivan G. Gomez^7^, Kohei Uchimura^8^, Benjamin D. Humphreys^8^, Chul Woo Yang^2^, Jae Yeon Lee^9^, Jin Kim^1^, Dong Woo Cho^9,^*, Benjamin S. Freedman^7,^* and Yong Kyun Kim^1,2,10,*^

^1^Cell Death Disease Research Center, College of Medicine, The Catholic University of Korea, Seoul, Korea

^2^Department of Internal Medicine, College of Medicine, The Catholic University of Korea, Seoul, Korea

^3^Integrative Research Support Center, College of Medicine, The Catholic University of Korea, Seoul, Korea

^4^Cancer Research Institute, College of Medicine, The Catholic University of Korea and Department of Medical Informatics, College of Medicine, The Catholic University of Korea

^5^Department of Biomedicine & Health Sciences, College of Medicine, The Catholic University of Korea

^6^Catholic iPSC Research Center, and Division of Rheumatology, Department of Internal Medicine, College of Medicine, The Catholic University of Korea, Seoul, Republic of Korea

^7^Division of Nephrology, Kidney Research Institute, and Institute for Stem Cell and Regenerative Medicine, Department of Medicine, University of Washington School of Medicine, Seattle, WA, USA.

^8^Division of Nephrology, Department of Medicine, and Department of Developmental Biology, Washington University School of Medicine, St. Louis, MO, USA

^9^Department of Mechanical Engineering, Pohang University of Science and Technology (POSTECH), Pohang, Kyungbuk, South Korea

^10^Department of Internal Medicine, College of Medicine, The Catholic University of Korea, St. Vincent’s Hospital, Suwon, Korea”

The authors apologize for any inconvenience caused.

