# Supplementary material for: Graft immaturity and safety concerns in transplanted human kidney organoids
Source: Exp Mol Med. 2019 Nov 28;51(11):1–13. doi: 10.1038/s12276-019-0336-x (PMC6881327; doi:10.1038/s12276-019-0336-x)
Supplement: Supplementary file 1 — Supplemental material [file 12276_2019_336_MOESM1_ESM.pdf]

Supplemental Figure 1. Structural changes in nephron-like structures after transplantation. **(A):** Representative confocal fluorescence images showing podocyte (NPHS1) and parietal epithelial cells (Claudin 1) populations in kidney organoids *in vitro* and the transplanted kidney organoids. Scale bars, 50μm. **(B):** Quantification of diameter of Bowman's space (kidney organoids in vitro (n=12) vs. transplanted kidney graft (n=3). *, $p < 0.05$) **(C)**: Representative confocal fluorescence images showing proximal tubular epithelial cells (LTL) populations in kidney organoids *in vitro* and the transplanted kidney organoids. Scale bars, 50μm. **(D):** Quantification of diameter of lumens of tubule-like structures (kidney organoids in vitro (n=12) vs. transplanted kidney graft (n=3). *, $p < 0.05$)

Supplemental Figure 2. Alternative differentiation protocols do not produce more mature cells. **(A):** Representative confocal immunofluorescence images from two additional batches of organoids distinct from the batch shown in Figure 5 for the Morizane and Takasato protocols, scaled identically to those images. **(B)**: Identical images as those shown in Figure 5A, with the scaling increased to the point of being overexposed in the tissue sections. Scale bars, 50 μm.

Supplemental Figure 3. Outgrowth of kidney organoids 7 days after implantation on glass plates coated with 3 % GelTrex. Scale bars, 600μm.

Supplemental Figure 4. Representative images of the transplanted kidney organoids graft at 2 weeks and 6weeks after renal subcapsular transplantation on NOD-SCID mouse kidney. Open arrows and black arrows indicate the kidney organoids graft at 2 weeks and 6weeks after transplantation, respectively. Scale bars, 2mm.

Supplemental Figure 5. (A): Representative images of H&E staining and immunohistochemical staining for safranin O of the kidney organoids transplanted with kidney dECM at 6 weeks after transplantation. Scale bars, 500 μm. **(B)**: Quantification of diameter of cartilages at 6 weeks after

transplantation. (transplanted kidney organoids (n=6) vs. transplanted kidney organoids with kidney dECM (n=6). *, $p < 0.05$).

Supplemental Figure 1

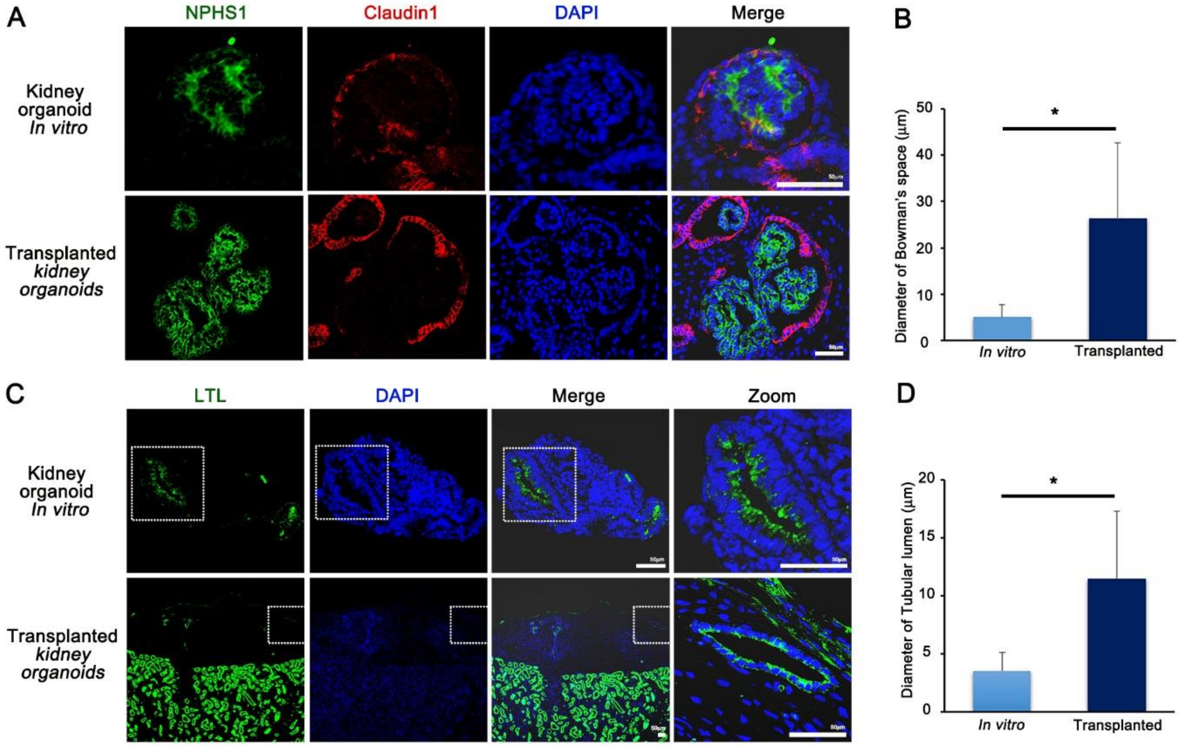

Supplemental Figure 2

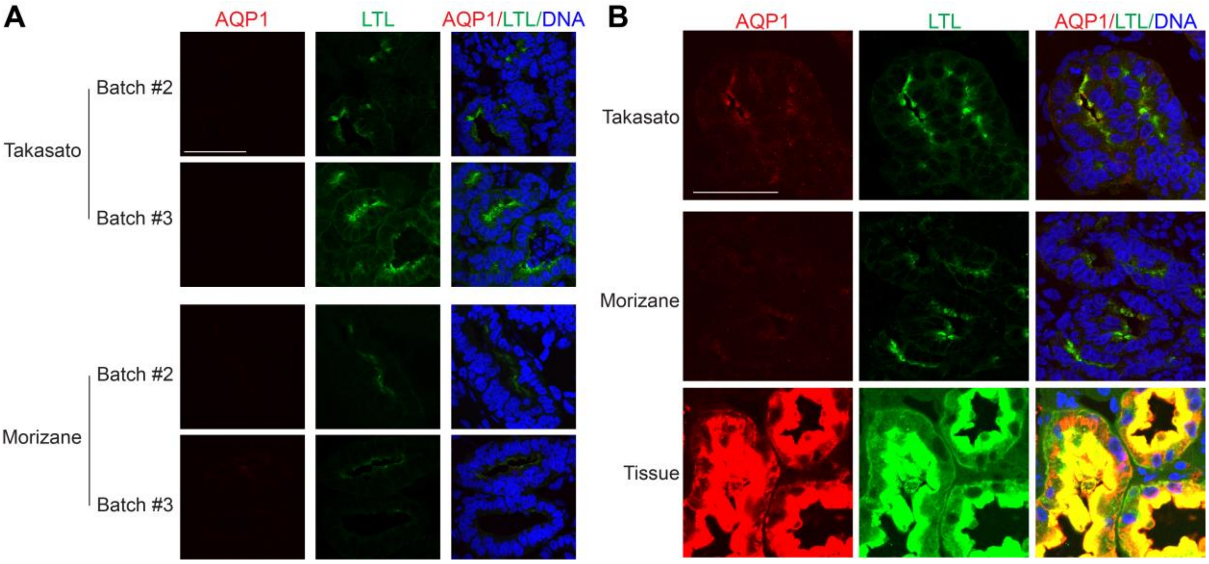

Supplemental Figure 3

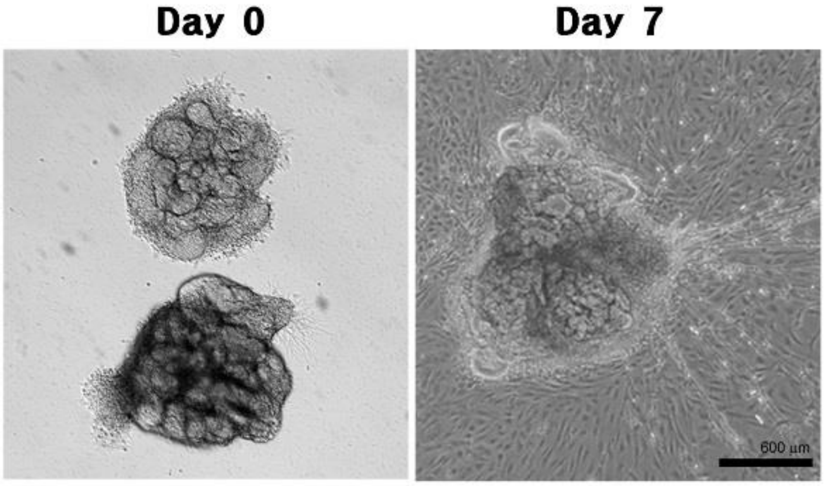

Supplemental Figure 4

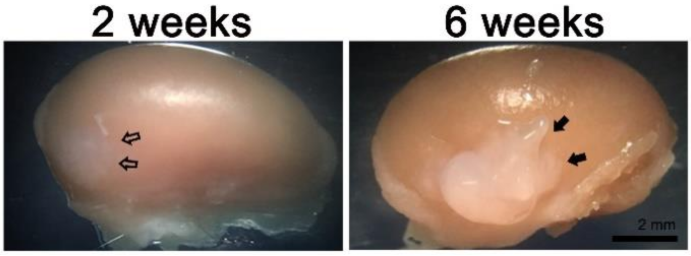

Supplemental Figure 5

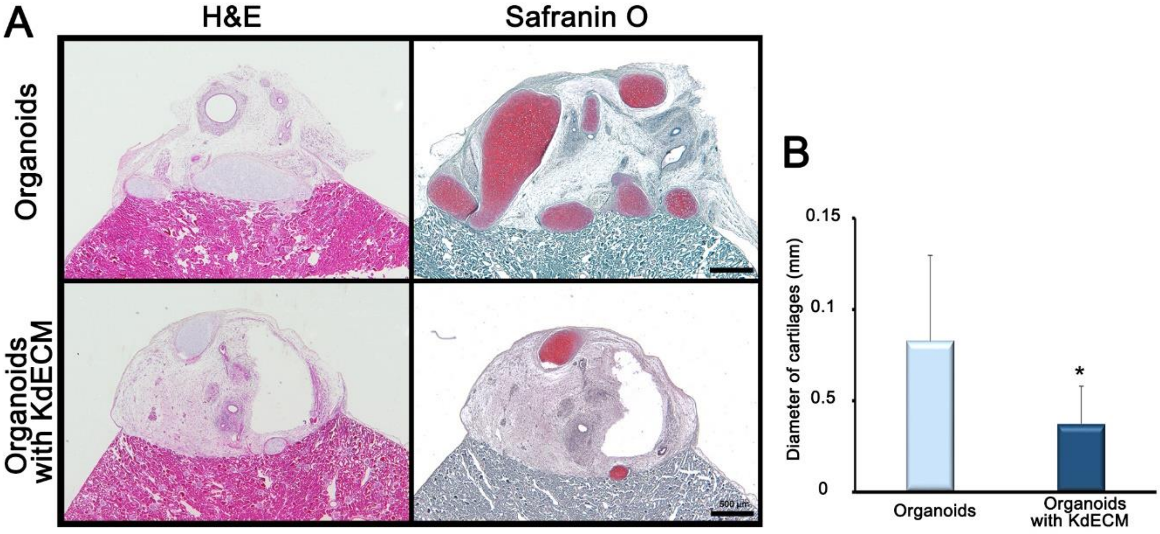

Supplemental Table 1. Top 20 down-regulated leading-edge genes in subset of cell cycles

Gene Symbol	Gene Name
HIST1H2AJ	histone cluster 1 H2A family member j
DBF4	DBF4 zinc finger
CDK1	cyclin dependent kinase 1
HIST1H2BH	histone cluster 1 H2B family member h
ZWILCH	zwilch kinetochore protein
DNA2	DNA replication helicase/nuclease 2
PLK4	polo like kinase 4
CCNB2	cyclin B2
KIF18A	kinesin family member 18A
MCM10	minichromosome maintenance 10 replication initiation factor
CDC6	cell division cycle 6
ORC6	origin recognition complex subunit 6
CCNB1	cyclin B1
NEK2	NIMA related kinase 2
E2F3	E2F transcription factor 3
NPM1	nucleophosmin 1
CENPN	centromere protein N
CENPH	centromere protein H
NDC80	NDC80, kinetochore complex component
CDC7	cell division cycle 7

Supplemental Table 2. Top 20 up-regulated leading-edge genes in subset of extracellular matrix organization

Gene Symbol	Gene Name
COL12A1	collagen type XII alpha 1 chain
MMP7	matrix metallopeptidase 7
COL14A1	collagen type XIV alpha 1 chain
COL15A1	collagen type XV alpha 1 chain
COL8A1	collagen type VIII alpha 1 chain
MMP13	matrix metallopeptidase 13
COL3A1	collagen type III alpha 1 chain
COL5A1	collagen type V alpha 1 chain
COL21A1	collagen type XXI alpha 1 chain
COL6A2	collagen type VI alpha 2 chain
MMP8	matrix metallopeptidase 8
MMP3	matrix metallopeptidase 3
COL1A1	collagen type I alpha 1 chain
COL16A1	collagen type XVI alpha 1 chain
COL19A1	collagen type XIX alpha 1 chain
CTRB1	chymotrypsinogen B1
TPSAB1	tryptase alpha/beta 1
TLL1	tolloid like 1
ADAMTS2	ADAM metallopeptidase with thrombospondin type 1 motif 2
COL4A4	collagen type IV alpha 4 chain
